# South African experts comment on FDA approval of dabigatran inatrial fibrillation

**Published:** 2011

**Authors:** Klug E, Bryer A, Aalbers J

The US Food and Drug Administration (FDA) Cardiovascular and Renal Drugs Advisory Committee voted 9 to 0 in favour of recommending dabigatran etexilate, an oral direct thrombin inhibitor for stroke prevention in patients with non-valvular atrial fibrillation (AF).

The FDA approved the standard dose of 150 mg bid (as also submitted by Boehringer Ingelheim in the European Union) without dose adjustments for tested p-GP inhibitors or for moderately renal-impaired patients.

No approval was given for the 110-mg strength; the justification seems to be that FDA felt the evidence was better for the 150-mg dose. However, the approval of the 75-mg strength for patients with severe renal impairment (15–30 ml/min) was driven by the FDA, based on criteria modelling.

Boehringer Ingelheim (BI) SA commented that the omission of the 110-mg dose came as a surprise and BI strongly supports the market need for both doses to be made available so physicians will be able to tailor therapy.

Interestingly, the Canadian health authority has approved dabigatran for the prevention of stroke and systemic embolism in patients with atrial fibrillation in whom anticoagulation is appropriate, marking the second approval of this new oral anticoagulant following the recent marketing authorisation by the FDA.

The Health Canada approval makes dabigatran etexilate available to AF patients in Canada in whom anticoagulation is appropriate, with the flexibility of two dosing regimens. A 150-mg bid dose is approved for the prevention of stroke and systemic embolism in patients with AF, and a 110-mg bid dose is specifically recommended for patients aged 80 years or older and is also available for patients at higher risk of bleeding.

## Comment from Dr Eric Klug, Sunninghill Hospital, Johannesburg

This is an exciting announcement; for the first time, an alternative to warfarin/coumadin in the anti-thrombotic management of atrial fibrillation. It was a surprise that approval did not include the 110-mgdose. The FDA clearly felt that the 150-mgdose found to be superior to warfarin should be used but added approval for the necessary lower safe dose (75 mg) for renal dysfunction.

## Comment from Prof Alan Bryer, Head:Division of Neurology and Stroke Unit,Groote Schuur Hospital and University of Cape Town; Chairman: South African Stroke Society

The advent of the direct thrombin antagonists such as dabigatran represents a major breakthrough in the prevention of stroke in patients with atrial fibrillation. In most parts of the world, the standard treatment for prevention of stroke in patients with atrial fibrillation is warfarin. However,treatment with warfarin is associated with a number of disadvantages, including an unpredictable response with a narrow therapeutic window that requires routine anticoagulation monitoring and often, frequent dose adjustments.

Warfarin also has a slow onset of action and takes about a week to reach an optimal INR, and similarly has a slow offset of action. A significant percentage of patients do not achieve optimal anti-coagulation within the ideal therapeutic window and many patients discontinue therapy. There are also numerous drug interactions with warfarin therapy.

Dabigatran, which has been approved by the FDA for secondary prevention of stroke in patients with atrial fibrillation, has a rapid onset of action and patients are fully anti-coagulated within 36 hours. Similarly, it has a rapid offset of action with normal coagulation after 48 hours following discontinuation. It has predictable anti-coagulant effects independent of factors such as age, body weight or race and has alow interaction rate with other drugs. There is no requirement to monitor coagulation,which is a major advantage for patients who have to take this drug.

The results of the much-awaited RE-LY trial have recently been published. In a population of patients with atrial fibrillation and a risk of stroke, the mean observation time in this study was two years (18 113 patients randomly assigned to fixed doses of dabigatran or adjusted dose warfarin). For the primary end poin tof stroke (ischaemic and haemorrhagic stroke) and systemic embolism, the higher dose of dabigatran (150 mg twice daily)demonstrated superior efficacy and a similar rate of major bleeds compared to warfarin.The lower dose of dabigatran (110 mg twice daily) was shown to cause less bleeding and was as effective as warfarin.

Prof Hans-Christoph Diener, a member of the steering committee of this trial, presented data from the trial at the World Stroke Congress in Seoul on 14 October 2010. A sub-study of those patients whohad had a prior transient ischaemic attack(TIA) or stroke before being randomised in the RE-LY trial were analysed and this sample represented 20% of the overall population of the study.

The results in this sub-group were identical to those of the overall trial. A complicationt hat is most feared in patients receiving anti-coagulation is cerebral bleeds and parenchymal haemorrhage and this was reduced in the two groups of patients taking either dose of dabigatran, compared with those on warfarin who had had a prior TIA or stroke before randomisation (considered secondary prevention). Although th egroup of patients with prior TIA or stroke represented only 20% of the total sample of 18 113 patients, both doses of dabigatran were shown to be non-inferior to warfarin but with less major haemorrhage.

These interesting results will no doubt be explored in further studies, but the introduction of the direct thrombin antagonists are undoubtedly the beginning of a new erain stroke prevention in patients with atrial fibrillation. However, in South Africa and other developing countries, where more than 80% of the country’s population is dependent on the public sector for health care,the cost of dabigatran and other similar drugs in development is likely to determine whether or not these drugs will replace warfarin in the prevention of stroke in patients with atrial fibrillation.

